# The Genus *Alistipes*: Gut Bacteria With Emerging Implications to Inflammation, Cancer, and Mental Health

**DOI:** 10.3389/fimmu.2020.00906

**Published:** 2020-06-09

**Authors:** Bianca J. Parker, Pamela A. Wearsch, Alida C. M. Veloo, Alex Rodriguez-Palacios

**Affiliations:** ^1^Department of Pathology, Case Western Reserve University School of Medicine, Cleveland, OH, United States; ^2^Department of Medical Microbiology, University Medical Centre Groningen, University of Groningen, Groningen, Netherlands; ^3^Division of Gastroenterology and Liver Disease, Case Western Reserve University School of Medicine, Cleveland, OH, United States; ^4^Germ-Free and Gut Microbiome, Digestive Health Research Institute, Case Western Reserve University, Cleveland, OH, United States

**Keywords:** *Alistipes finegoldii*, inflammatory bowel diseases, *A. putredinis*, *A. onderdonkii*, *A. shahii*, *A. indistinctus*, *A. senegalensis*, *A. timonensis*

## Abstract

*Alistipes* is a relatively new genus of bacteria isolated primarily from medical clinical samples, although at a low rate compared to other genus members of the *Bacteroidetes* phylum, which are highly relevant in dysbiosis and disease. According to the taxonomy database at The National Center for Biotechnology Information, the genus consists of 13 species: *Alistipes finegoldii, Alistipes putredinis, Alistipes onderdonkii, Alistipes shahii, Alistipes indistinctus, Alistipes senegalensis, Alistipes timonensis, Alistipes obesi, Alistipes ihumii, Alistipes inops, Alistipes megaguti, Alistipes provencensis*, and *Alistipes massiliensis. Alistipes communis* and *A. dispar*, and the subspecies *A. Onderdonkii* subspecies vulgaris (vs. *onderdonkii* subsp.) are the newest strains featured outside that list. Although typically isolated from the human gut microbiome various species of this genus have been isolated from patients suffering from appendicitis, and abdominal and rectal abscess. It is possible that as *Alistipes* spp. emerge, their identification in clinical samples may be underrepresented as novel MS-TOF methods may not be fully capable to discriminate distinct species as separate since it will require the upgrading of MS-TOF identification databases. In terms of pathogenicity, there is contrasting evidence indicating that *Alistipes* may have protective effects against some diseases, including liver fibrosis, colitis, cancer immunotherapy, and cardiovascular disease. In contrast, other studies indicate *Alistipes* is pathogenic in colorectal cancer and is associated with mental signs of depression. Gut dysbiosis seems to play a role in determining the compositional abundance of *Alistipes* in the feces (*e.g*., in non-alcoholic steatohepatitis, hepatic encephalopathy, and liver fibrosis). Since *Alistipes* is a relatively recent sub-branch genus of the *Bacteroidetes* phylum, and since *Bacteroidetes* are commonly associated with chronic intestinal inflammation, this narrative review illustrates emerging immunological and mechanistic implications by which *Alistipes* spp. correlate with human health.

## Introduction

The human gut microbiome is acquired at birth and as individual go through life, their gut becomes a home to trillions of microorganisms, namely bacteria. With the use of 16S rRNA gene sequencing a diverse profile of bacterial phyla has been identified, with the two most common being *Bacteroidetes* and *Firmicutes*. Collectively, the composition of the gut microbiota amounts to a massive number of cells and functions that equate (even surpass) to that of an additional organ (1). The human microbiota plays a critical role in regulation of the immune response, protection against pathogens, aiding in digestion, as well as in neurologic signaling and vascularization (2). Notably, the microbiota has been found to be involved in health and disease. The imbalance, or dysbiosis, of the intestinal microbiota has been attributed to numerous diseases such as cancer (3), cardiovascular disease (4), inflammatory bowel disease (5), and nervous system disorders (6). Investigators have examined various genera and species that make up the microbiome trying to decipher their role as a collective trying to identify individual microbial species with the ability to modulate diseases.

Taxonomically, *Alistipes* (a. li. sti'pes) is a genus described in 2003 after being discovered in tissue samples of children with appendicitis (7). *Alistipes* are anaerobic bacteria found mostly in the healthy human gastrointestinal (GI) tract microbiota (8). According to the taxonomy database at The National Center for Biotechnology Information (NCBI, txid239759), as of April 2020, *Alistipes* consists of 13 species: *Alistipes finegoldii, Alistipes putredinis, Alistipes onderdonkii, Alistipes shahii, Alistipes indistinctus, Alistipes senegalensis, Alistipes timonensis, Alistipes obesi, Alistipes ihumii, Alistipes inops, Alistipes megaguti, Alistipes provencensis*, and *Alistipes massiliensis* (the latter strain reported as “unpublished by Lacroix et al., as of March 4, 2004, NCBI:txid265312” remains unpublished under that name). However, a case study in 2017 from the same group of scientists reported a novel species called *Tidjanibacter massiliensis* Marseille-P3084, isolated from the colon of a person with irritable bowel syndrome, with 92.1% sequence homology to *A. putredinis* (9), but the strain has not been officially recognized or deposited in public strain biorepositories. To date it is unclear if the *Tidjanibacter* is the same *Alistipes* isolate, or what occurred with the publication of the *Alistipes massiliensis* for which there is no genome or 16S RNA gene sequences available in NCBI. Three newer species were added to the the “List of Prokaryotic names with Standing in Nomenclature” database in January 2020: *A. communis* (strain 5CBH24; DSM 108979; JCM 32850), *A. dispar* (5CPEGH6; DSM 108978; JCM 32848), and the sub-speciation scheme for *A. Onderdonkii* as subsp. *vulgaris* subsp. nov. and the subsp. *onderdonkii* subsp. nov. (https://lpsn.dsmz.de/genus/alistipes, which listed only 11 species on May 12, 2020) (10).

Anecdotally, the first species of *Alistipes* to be discovered was *A. finegoldii* (fi.ne.gol'di.i) which was then named after Sydney M. Finegold (born in 1921; died on September 27, 2018, age 97), a contemporary American researcher in anaerobic bacteriology and infectious diseases clinician who held several Emeritus academic positions at the UCLA and the Wardsworth VA Hospital in Los Angeles since the year 2000 (7, 11). Several recent articles commemorate Dr. Finegold's career in anaerobic microbiology, who greatly contributed to our understanding of anaerobes, which led to the species of *Alistipes* being named after him (12). With a remarkable productivity, Dr. Finegold left a legacy with 819 publications on various aspects of medicine and anaerobic microbiology (13). Alongside *A. finegoldii*, in 2003, *Bacteroides putredinis* was reclassified as *A. putredinis* based on 16S rRNA gene sequencing and biochemical features that showed this species did not belong to the genus *Bacteroides*, but to the genus *Alistipes* (7). Dr. Finegold's charisma is illustrated with the species *A. onderdoinklii*, which was named to honour Andrew B. Onderdonk, a contemporary American microbiologist, Professor of Pathology at Harvard Medical School, for his contribution to intestinal and anaerobic microbiology.

In recent years, several studies have investigated the alterations in bacterial abundance for *Alistipes* in human patients and mouse models during disease. Studies have shown that *Alistipes* dysbiosis can be either beneficial, or harmful. *Alistipes* has been implicated in liver fibrosis (14), colorectal cancer (15), cardiovascular disease (16), and mood disorders (17), among other potential diseases. Additionally, the unique way of fermenting amino acids, putrefaction, has implicated *Alistipes* to play a critical role in inflammation and disease (18). The objective of this review is to expound upon the relationship between *Alistipes* and several non-communicable diseases and to highlight the implications of *Alistipes* dysbiosis.

## Phenotyping, Culture, and Speciation

*Alistipes* is classified as gram-negative, rod-shaped, anaerobic, and non-spore forming. The species within this genus are non-motile except for *A. obesi*. The genus consists of 12-published species (*A. massiliensis* is not yet published, see above). *Alistipes finegoldii* (type strain AHN 2437^T^; CCUG 46020) has circular colonies (0.3–1.0 mm; raised, gray to opaque on sheep blood agar). Despite supplementation, its growth is difficult in liquid media. *Alistipes finegoldii* is bile-resistant and esculin-negative. This bacterium is catalase-negative and nitrogen-reductase negative; and it cannot liquefy gelatin in liquefaction test. This bacterium can hydrolyze tryptophan to indole. In peptone yeast glucose (PYG) broth the major acid produced is succinic acid with a minor amount of acetic and propionic acid produced. The main fatty acid is 13-metyltetradecanoic acid (iso-C15:0). These strains of *A. finegoldii* are resistant to vancomycin, kanamycin, and colistin, as it is expected for *Bacteroides*, according to the Wadsworth manual (19) (named after the hospital where Dr. Finegold worked as a physician and scientist). For the primary identification of anaerobic bacteria, the further use of a bile susceptibility test combined with the antimicrobial resistance helps in differentiating *Alistipes* species as illustrated for some species below (for comparison, *Bacteroides* are resistant to bile) (20), although the precise definition of a species is a subject of constant debate (21). The phenotypic characteristics of 10 *Alistipes* spp. in culture media are summarized in Table 1.

**Table 1:** Summary of first 10 *Alistipes* species and their characteristics.

	***A. finegoldii:*** human appendiceal tissue	***A. putredinis:*** feces, appendicitis, abdom/rectal abscess foot rot sheep, farm soil	***A. onderdonkii:*** abdominal abscess	***A. shahii:*** appendix tissue, urine	***A. indistinctus:*** human feces	***A. timonensis:*** feces, healthy patients Senegal	***A. senegalensis:*** feces, healthy patients Senegal	***A. obesi:*** fecal flora of a French patient suffering from obesity	***A. ihumii:*** fecal flora of female French patient; severe anorexia nervosa	***A. inops:*** humanfeces
Gram stain	–	–	–	–	–	–	–	–	–	–
Motile	–	–	–	–	–	–	–	+	–	–
Bile resistant	+	–	+	+	–	/	/	/	/	/
Pigment	+	–	+	+	–	+	+	/	/	/
Esculin	–	/	/	/	/	/	/	/	/	/
Gelatin	+	/	/	/	/	/	/	/	/	/
Catalase	–	+	–	–	+	+	+	+	–	–
Nitrogen red.	–	–	–	–	–	/	/	–	–	–
Indole	+	+	+	+	–	+	+	–	–	+
Fermentative	F	NF	F	F	/	NF	F	NF	/	NF
Urease	/	–	–	–	–	/	/	–	–	–

*Antibiotic Resistance. A. finegoldii, vancomycin, kanamycin, and colistin. A. putredinis, his strain is sensitive to clindamycin, cefoxitin, chloramphenicol, erythromycin, and metronidazole and moderately resistant to tetracycline and doxycycline. A. timonensis, susceptible to penicillin G, amoxicillin + clavulanic acid, imipenem and clindamycin; A. timonensis is susceptible to metronidazole. A. senegalensis, susceptible to penicillin G, amoxicillin + clavulanic acid, imipenem and clindamycin; A. senegalensis is resistant to metronidazole. A. obesi, susceptible to imipenem, ciprofloxacin, metronidazole, nitrofurantoin, and rifampicin, but resistant to penicillin G, amoxicillin, amoxicillin-clavulanic acid, erythromycin, vancomycin, gentamicin, doxycycline, ceftriaxone, and trimethoprim/sulfamethoxazole. A. ihummi, susceptible to amoxicillin, imipenem, and clindamycin, but resistant to vancomycin. /, not reported; F, fermentative; NF, non-fermentative*.

From a clinical perspective, it is reasonable to expect that bile-resistant strains are more likely to be present or abundant than bile-susceptible strains in the terminal ileum where most bile reabsorption takes place in several mammalian species including humans (22) and where chronic inflammatory bowel conditions are more likely to occur due to alterations in bile-mediated T-cell immunoreactivity (23). However, with the recent discovery of this genus, and the emerging complexity of the genus speciation, it will be necessary to determine if the resistance to bile acids indeed determines the *Alistipes* abundance regionally within the GI, or clinically in diseases characterized by alterations in bile production, which originates in the liver, or their recirculation pathways through the small intestine. Also, clinically relevant, some strains produce beta-lactamases (7), which enhance their antimicrobial resistance to beta-lactam antibiotics including penicillin and cephalosporins.

The species *A. putredinis* (type strain ATCC 29800^T^) has been isolated from various specimens such as feces, appendiceal tissue of a patient with acute appendicitis, abdominal and rectal abscesses, foot rot in sheep, and even farm soil (7). This species has circular to slightly irregular convex colonies (pinpoint to 0.5 mm; smooth, translucent to gray). This is a non-pigment producing species and is not 20% bile-resistant. It is catalase-positive and nitrate reductase- and urease-negative. This bacterium can hydrolyze tryptophan to indole. This species is non-fermentative. In 6-day old PYG broth only trace amount of acid was produced, however, in 24-h chopped meat carbohydrate broth succinic acid was produced as a major acid product with minor amounts of acetic, isobutyric, isovaleric, and propionic acid. This strain is sensitive to clindamycin, cefoxitin, chloramphenicol, erythromycin, and metronidazole and moderately resistant to tetracycline and doxycycline (7).

*Alistipes onderdonkii* (type strain WAL 8169^T^; CCUG 48946^T^; ATCC BAA-1178^T^) and *A. shahii* (type strain WAL 8301^T^; CCUG 48947^T^; ATCC BAA-1179^T^) were isolated from abdominal abscess and appendix tissue respectively, as well as urine (24). They have circular colonies (0.5–0.8 mm and 0.5–1 mm, respectively; gray to opaque on blood agar) and are pigment producing, non-fluorescing when grown on rabbit blood agar. Both species are resistant to 20% bile and hydrolyze tryptophan to indole. They are catalase-, nitrogen reductase-, and urease-negative. Mannose and raffinose are fermented when using the API rapid ID 32A system. Succinic acid is the major metabolic end-product with minor production of acetic and propionic acid. The primary long-chain fatty acid is iso-C15:0 (24). Details for *A. onderdonkii* subspeciation as *vulgaris* subsp. nov. (3BBH6T = JCM 32839T = DSM 108977T, isolated from human feces) for can be found at https://lpsn.dsmz.de/genus/alistipes.

*Alistipes indistinctus* (type strain YIT 12060^T^; DSM 22520^T^; JCM 16068^T^) was the next species to be discovered. It was isolated from human feces. This species is slightly different, since its shape is more coccoid than rod-shaped, as well as its inability to hydrolyze tryptophan to indole. Its colonies are circular (0.1–0.5 mm slight opaque and gray on modified GAM agar). This bacterium is susceptible to 20% bile, catalase-positive, and urease- and nitrogen reductase-negative. The major products in PYG broth are succinic and acetic acid. The major cellular fatty acid is iso-C15:0 (25).

Two other species, *A. senegalensis* (type strain JC50^T^; CSUR P156; DSM 25460) and *A. timonensis* (type strain *JC136*^*T*^*; CSUR P148; DSM 25383)*, were initially isolated from the fecal flora of healthy patients in Senegal. They have circular colonies (0.2–0.3 mm) and are pigment producing. They are catalase and can hydrolyze tryptophan to indole. *A. senegalensis* does ferment mannose, and *A. timonensis* does not. These strains of bacteria are susceptible to penicillin G, amoxicillin plus clavulanic acid, imipenem, and clindamycin. Furthermore, *A. senegalensis* strains are resistant to metronidazole and *A. timonensis* strains are susceptible to metronidazole (21, 26).

*Alistipes obesi* (type strain ph8^T^; CSUR; P186;DSM 25724) was isolated from the fecal microbiota of a French patient suffering from obesity (27). It is a pigment producing species with colonies that are 0.5 mm in diameter; translucent and light gray on blood enriched Columbia agar. It is catalase-positive, nitrogen reductase-, and urease-negative as well as non-fermentative. This bacterium cannot hydrolyze tryptophan to indole. These strains are susceptible to imipenem, ciprofloxacin, metronidazole, nitrofurantoin, and rifampicin, but resistant to penicillin G, amoxicillin, amoxicillin-clavulanic acid, erythromycin, vancomycin, gentamicin, doxycycline, ceftriaxone, and trimethoprim/sulfamethoxazole (27).

*Alistipes ihumii* (type strain AP11^T^; CSUR P204; DSM 26107) was isolated from the fecal microbiota of a female French patient suffering from severe anorexia nervosa. This species forms colonies that are 0.2 mm and translucent on blood-enriched Columbia agar. This bacterium is non-fermentative. It is negative for urease, nitrate reduction, and catalase activity. This bacterium cannot hydrolyze tryptophan to indole. *A. ihumii* strains are susceptible to amoxicillin, imipenem, and clindamycin, but resistant to vancomycin (28).

The species *Alistipes inops* (type strain 627^T^; 5DSM 28863^T^; VKM B-2859^T^) was isolated from human feces. The colonies are circular (0.18–0.30 mm; light gray, with a pale brown center). It has scanty growth on liquid media. It is positive for indole production and negative for catalase, nitrate reductase, and urease. This bacterium is non-fermentative. The major metabolic end products in PYG broth are succinic and acetic acid. The major fatty acid is iso C15:0 (8).

Recently, between November 2019–January 2020, four, new species of *Alistipes* were discovered, *A. megaguti, A. provencensis, A. communis* (type strain 5CBH24; DSM 108979; JCM 32850, commonly in human feces) and *A. dispar* (5CPEGH6; DSM 108978; JCM 32848, from human feces). *A. megaguti* (type strain Marseille-P5997^T^) was isolated from a fresh fecal sample of a young healthy female. This species forms colonies that are 0.2–0.9 mm in diameter on blood-enriched Columbia agar. *A. megaguti* are catalase, urease, and oxidase negative. *A. provencensis* **(**type strain Marseille-P2431^T^) was isolated from a male patient with hypertension and diabetes. This species forms colonies that are 0.4–0.64 mm in diameter on blood-enriched Columbia agar. This species is oxidase and urease negative, and catalase positive (29).

**Figure 1:**
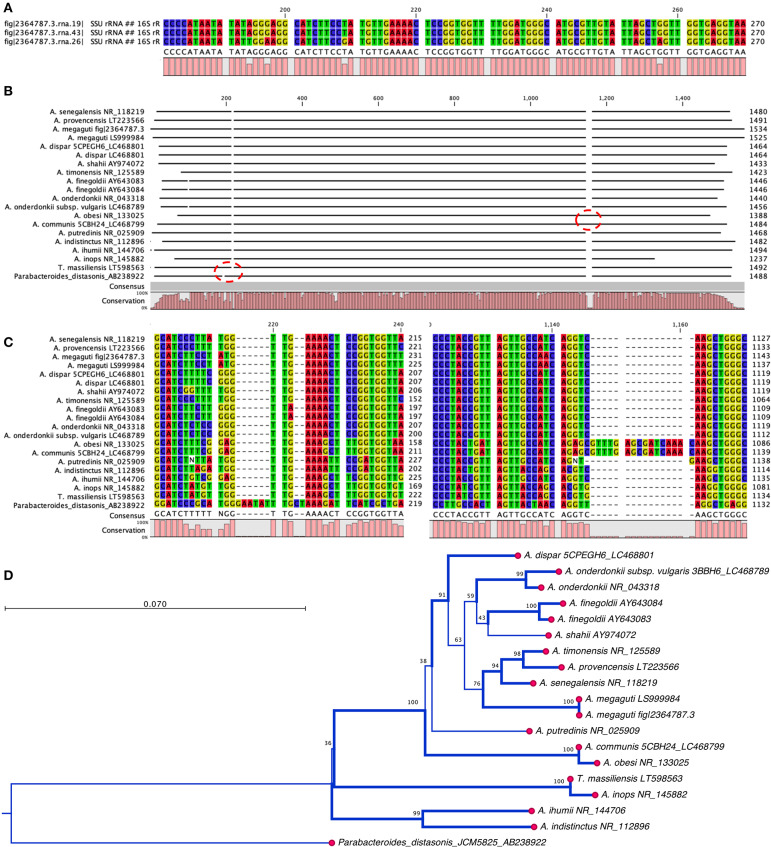
Nucleotide and phylogram of 16S rRNA gene DNA sequences from thirteen strains described in the genus *Alistipes*. **(A)** *Alsitipes megaguti* complete 16S rRNA gene sequences derived from PATRIC complete genome illustrates slight differences between three gene copies contained within three operons in a single genome. Only the most divergent parts of the genes are shown. **(B)** Overview of complete alignment for the 13 strains. Notice the *A. obesi* insertion at around 1,100 bp position. **(C)** Detail of sequence insertion in the *A. obesi* starting at position 1,137 bp position. **(D)** Overview of the distance tree with 10,000 bootstrap branch values depicted if value >90%. Branching set at 60% threshold. Notice that differences may occur in part due to uneven partial gene sequences. Analysis conducted with CLC genomics viewer. Notice position of *T. massiliensis* vs. *A. inops* and *A. putredinis*.

The phenotypic identification of *Alistipes* species is feasible (Table 1), yet challenging. A more suitable way to identify *Alistipes* spp. is via NGS (next generation sequencing-based microbiome studies) (30). However, most studies for the classification of new species, which cannot be fully sequenced have been based on Sanger sequencing of the full gene or parts of the 16S rRNA gene. For reference, the specificity of various primers employed for the identification of *Alistipes* species are listed and referenced in Table 2, while the details in nucleotide sequence homology and genetic distances within the genus are depicted in Figure 1. Compared to culture techniques, the NGS method is more powerful for bacterial identification. However, it does not provide information on the metabolic functions of the organism. Another form of bacterial identification is through metagenomic studies. In this approach, bacterial DNA is processed and sequenced along with host DNA. A new clinical approach that is faster and more effective than Sanger sequencing for bacterial classification is based on mass-spectrometry. Specifically, new rapid high-through put strategies based on matrix assisted laser desorption/ionization time-of-flight mass spectrometry (MALDI-TOF MS) have become widely adapted and common in clinical microbiological settings, which require the constant update of databases to incorporate the spectral of new bacterial strains, primarily of clinical relevance.

**Table 2:**
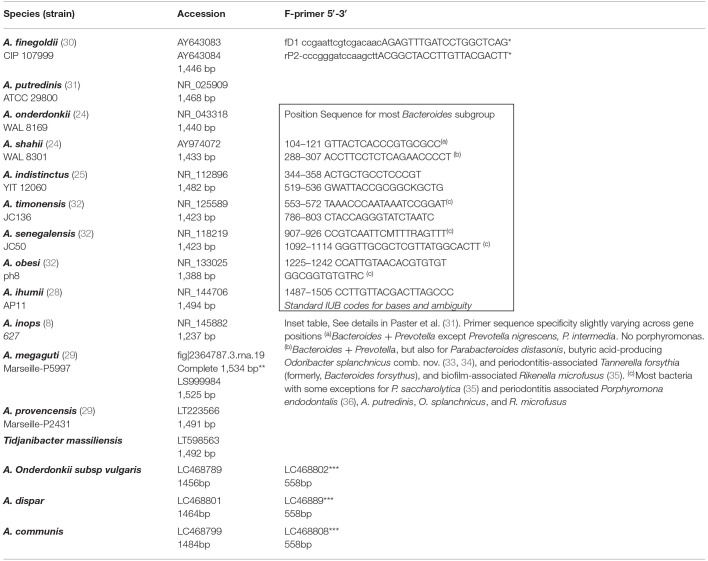
Referent partial Sanger 16S rRNA gene sequences and primers for *Alistipes* spp.

*Primers (upper case) used by Weisburg et al. (37) and designed for most eubacteria. f, forward; r, reverse; D, distal; P, proximal. Linker sequences containing restriction sites for cloning are designated in lower case. The “f” series of linkers contain EcoRI and SalI sites, and the “r” series contain HindIJl, BamHI, and XmaI recognition sequences. Reverse primers produce sequences complimentary to the rRNA. Primers rPl, rP2, and rP3 are identical except for the 17th base from the 3′ end. The P2 primer was determined as the sequence providing the greatest diversity of bacteria. FASTA files available for download at https://www.ncbi.nlm.nih.gov/search/all/?term=NR__125589^**^complete sequence extracted from complete genome available in PATRIC https://docs.patricbrc.org.

***Phylogenetic analysis for speciation can also be conducted for Alistipes using the heat shock protein 60 gene (hsp60) as reported by Sakamoto in 2020. Accession numbers for the whole genome sequences of strains 3BBH6T, 5CBH24T, 5CPEGH6T, 5CPYCFAH4, 5NYCFAH2 and 6CPBBH3 are AP019734-AP019739, respectively.

## Mass Spectroscopy for Identification of Bacterial Species

Since the innovative use of MALDI-TOF MS to expedite the identification of microbes in clinical microbiology, there has been a major increase in our understanding of the presence and prevalence of *Alistipes* spp. in clinical medicine. MALDI-TOF MS is a method based on protein spectra, enabling identification of an unknown bacterium within minutes. Due to the introduction of this method in diagnostic microbiology laboratories, fastidious organisms which could previously only be identified using molecular methods, are now identified with ease. With the advancement of MALDI-TOF MS, there has been collaborative initiatives to improve microbial identification, including the European Network of Rapid Identification of Anaerobes (ENRIA), which is multi-national consortium of laboratories in Europe with the goal of unifying the MALDI-TOF MS database for the rapid identification of clinical anaerobes. According to the ENRIA validation study, conducted in 2018 (38), at present 5 *Alistipes* species are represented in the MALDI-TOF MS database: *A*. *finegoldii, A. onderdonkii, A. shahii, A. indistinctus*, and *A. putredinis*.

To contextualize the current relevance of the genus in clinical diagnostics, we conducted a screening of the number of *Alistipes* isolates recovered from human clinical samples at the University Medical Center Groningen, Groningen, the Netherlands, within the 7 years prior to submission of this manuscript. Keeping in mind that only species represented in MALDI-TOF MS databases have been identified using this method, it is noteworthy to mention that only 11 (*n* = 11) clinical isolates belonged to the *Alistipes* genus. Of relevance, these isolates were identified only as either *A. onderdonkii* (*n* = 7) or *A. finegoldii* (*n* = 4). The *Alistipes* isolates were recovered from pus, blood, ascites fluid or from an unspecified specimen. Considering that there have been at least 13 different species identified as distinct according to the NCBI lineage database (as of November 2019), it is interesting that only few species (*n* = 5) have been identified in clinical settings screened by ENRIA. The lack of identification of the other species (e.g., *A. senegalensis, A. timonensis, A. obesi, A. ihumii*, and *A. inops*) in this population indicated that either: (i) *Alistipes* may have specific patterns of geographically-restricted distribution, (ii) that their prevalence in the environment and patients is rather low, (iii) that they are comparatively more difficult to be cultured from clinical specimens using media that apparently facilitates the recovery of the other *Alistipes* spp. (namely, *A onderdonkii* and *A. finegoldii*), (iv) that the MS profile used to differentiate species is still suboptimal to discriminate such species as separate, or (v) that the original species descriptions may not be reproducible descriptions in the context of culture and mass spectrometry profiles.

## Ecology in Diseases

According to the NCBI, the full lineage of *Alistipes* is Bacteria; *Bacteroidetes*; *Bacteroidia*; *Bacteroidales*; *Rikenellaceae* (NCBI: txid239759). Therefore, *Alistipes* is a genus member of the *Rikenellaceae* family (NCBI:t xid171550), which is a small family within the *Bacteroidales* order that is composed of eight genera: *Acetobacteroides, Alistipes, Anaerocella, Millionella, Mucinivorans, Rikenella, Ruminofilibacter*, and *Tidjanibacter*. These genera are commonly found in the intestinal tract of various animals and humans, where they are believed to have symbiotic relationships with the host. One of the most commonly detected species from the genus *Alistipes*, that can be isolated from human feces, is *A. onderdonkii* (39).

From an ecological perspective, *Alistipes* is found primarily in the gut of healthy humans (8). However, *Alistipes* has also been isolated from the blood stream, as well as appendicular, abdominal, perirectal and brain abscesses highlighting their potential opportunistic pathogenic role in human diseases. *Alistipes* has been found in other body fluids, namely, urine and peritoneal fluid (8). Of contrasting interest within this genus, *A. ihumii* has been isolated from the feces of a patient suffering from anorexia nervosa, while *A. obesi* was isolated from a patient suffering from morbid obesity. Combining the later observations with the fact that *Alistipes finegoldii* has been considered a growth promoter in broiler chickens (40), and *A. putredinis* has been observed to increase with cruciferous vegetable intake in humans (41), it is reasonable to assume that different *Alistipes* species may have different roles in nutrition and health, depending on the host, and the bodily system influenced. Although the NCBI taxonomy database has a disclaimer stating that it is not an authoritative source for nomenclature or classification, the NCBI recommend the database users to consult the relevant scientific literature for the most reliable information, which is summarized below and in Table 3.

**Table 3:** Summary of studies reporting the experimental or observational associations between *Alistipes* spp. and various non-communicable diseases (2003–2019).

**Disease**	**Study model**	**Study design**	***Alistipes* effects**
Inflammation Colitis (42) (Protective)^*^	Mice: BALB/c	Oral DSS-colitis. 16S microbiome and oral infection.	Pglyrp1-4 (antibacterial immunomodulator gene) KO mice have less *Alistipes* spp. Oral *A. finegoldii* attenuates antibiotic > DSS-colitis in WT mice
Inflammation Liver cirrhosis (43) (Protective)	Humans: China	Meta-omics-based study analyzing urine and stool samples from health controls, compensated and decompensated LC patients	In both compensated and decompensated LC patients *A. shahii* and *A. putredinis* decrease and during progression from compensated to decompensated *A. indistinctus* further decreased
Inflammation Acute hepatic encephalopathy (44) (Protective)	Humans: Taiwan	Longitudinal cohort before treatment, 2–3 d after, and 2–3 mo after	*Alistipes* decreases during AHE compared to healthy controls, compensated, and decompensated cirrhosis. Also associated with recurrence at 1 year
Inflammation NASH/NAFLD (14) (Protective)	Humans: Germany	16S rRNA sequencing of stool	*A. finegoldii* decreased in NASH when compared to healthy controls. *A. onderdonkii* was reduced in patients with NAFLD
Inflammation Hepatocellular carcinoma, HCC (45) (Protective)	Mice: Male C57BL6/N	Metagenomic study using the stool of control mice vs. mice with tumor. The mice were fed probiotic [mixture (1:1:1) of *Lactobacillus rhamnosus* GG, *Escherichia coli* Nissle 1917 and heat inactive VSL#3]	*Alistipes* spp. and *A. shahii* increased in mice receiving the probiotic, in which there was modulation in pro-inflammatory cytokines in tumor microenvironment
*Cancer* Colorectal cancer (46) (Pathogenic)	Mice: C57BL/6J	qPCR/16S rRNA seq. to identify *Alistipes* in Lcn2^−/−^, Il10^−/−^ mice. Il10^−/−^ mice gavaged with *A. finegoldii* to determine tumor localization.	*Alistipes* promotes colorectal cancer via IL-6/STAT3 pathway causing right sided tumorigenesis
Cardiovascular Atrial fibrillation (16) (Protective)	Humans: China	Metagenomic and metabolomic analyses of fecal samples extracted from patients with non-vascular atrial fibrillation or HC	*Alistipes* decreases in abundance in atrial fibrillation group when compared to HC
Cardiovascular Hypertension (47, 48) (Pathogenic)	Humans: America	Metagenomics of DNA extracted from fecal samples. High blood pressure (HBP) vs. controls.	*A. finegoldii* and *A. indistinctus* increase in HBP; LPS increase, Th17 cells, and inflammation; in HBP decrease in butyrate-producing cells; increase plasma intestinal fatty acid binding protein (I-FABP); and gut epithelial tight junction Zonulin
Cardiovascular Congestive heart failure (49) (Protective)	Humans: China	Metagenomics of DNA extracted from fecal samples	*Alistipes* decreased together with *Faecalibacterium* and *Oscillibacter*, while *Ruminococcus, Acinetobacter*, and *Veillonella* increased
Cardiovascular atherosclerosis cardiovascular disease (50) (Protective)	Humans: China	Metagenomics of DNA extracted from fecal samples	*Alistipes shahii* decreases together with *Bacteroides* spp., *Prevotella copri*, in ACVD
Mental health Anxiety (17) (Pathogenic)	Mice: BALB/c	Stress induced by grid floor housing. DGGE,16S Microbiome, Triple test, tail suspension test, and burrowing	*Alistipes* spp. increased after 2 weeks on grid floor housing. There was also significant change in the mice behavior
Mental health Myalgic encephal. Chronic fatigue (51) (Pathogenic)	Humans: Belgium Norway	16S rRNA sequencing of stool.	3.8-fold increase of *Alistipes* spp. in Norwegian patients vs. Norwegian controls
Mental health depression (52) (Pathogenic)	Humans: China	Pyrosequencing of DNA from feces of either healthy controls of patients with active- and responded-major depressive disorder (MMD).	*Alistipes* increases in both A-MDD and R-MDD groups when compared to HC; due to being indole positive decrease in serotonin availability. *Faecalibacterium* decreases in MMD
Mental health Autism spectrum disorder (53) (Protective)	Humans: Italy	Pyrosequencing using 16S rRNA from stool samples of 40 autistic and 40 neurotypical pediatric patients	*Alistipes* decreased with *Bilophila, Dialister, Parabacteroides, Veillonella*. *Collinsella, Dorea Corynebacterium*, and *Lactobacillus* increased
Mental health PDD-NOS (autism) (54) (Pathogenic)	Humans: Italian children	bTEFAP analysis on DNA and cDNA samples from each patient and pyrosequencing of 16S rDNA and rRNA	*Alistipes* was found at the highest abundance in AD and PDD-NO; possibly due to high production of propionic acid which has been shown to have neurobiological effects in rats

* *Protective; overall interpretation of results with respect to outcome for each disease*.

## *Alistipes* in Liver Disease and Short-Chain Fatty Acids

Hepatocellular carcinoma (HCC) is the second deadliest form of cancer worldwide (55).

HCC is often developed from advanced liver fibrosis that is caused by cirrhosis, non-alcoholic fatty liver disease (NAFLD), or non-alcoholic steatohepatitis (NASH). These liver diseases have been associated with the “microbiota-liver axis,” indicating that dysbiosis as one of the potential causes (56). In studies done on the microbiota composition and liver fibrosis. It is seen that throughout the advancement of the fibrosis that *Alistipes* is decreased.

For example, in patients with compensated and decompensated liver cirrhosis (LC), a paired-end metagenomic sequence of the gut microbiome from fresh stool samples of healthy volunteers and patients with various types of LC. This study showed a decrease of *A. shahii* and *A. putredinis* when compared to healthy controls. This finding of an increase in the abundance of *Alistipes* in healthy control patients in comparison to patients with LC has also been seen in studies focusing on feces and biopsies of LC patients (57). Moreover, a decrease of *A. indistinctus* was observed as the disease progressed from compensated to decompensated (43). Once an individual has decompensated liver cirrhosis, the patient begins to generate a multitude of severe complications, such as hepatic encephalopathy. Another study showed that when comparing stool microbiome between patients suffering from decompensated liver cirrhosis with acute hepatic encephalopathy, *Alistipes* has a protective role and the decrease in its abundance correlates to an increase of hepatic encephalopathy recurrence (44). Thus, a decrease in *Alistipes* spp. correlates with the progression of liver cirrhosis into the decompensated state.

Additionally, the reduction in *Alistipes* abundance in patients with liver fibrosis can be seen in other fibrotic diseases such NASH and NAFLD. Rau et al. (14) showed that patients with NAFLD with substantial fibrosis had a reduction in fecal concentration of acetate and propionate, with no significant difference in butyrate concentration. When comparing healthy controls with patients suffering from NASH, there is a significant reduction in *A. finegoldii* with the normalized count mean reduced from 542 to 19 (fold-change of −1.829). Moreover, in NAFLD patients with significant fibrosis, a major reduction in *A. onderdonkii* was observed from 285 to 31 (fold change of −2.566). It is noteworthy that *Alistipes* spp. decreases in these advanced fibrotic patients as well as fecal acetate and propionate levels. This correlates with a study done by Polansky et al. (58) where they showed that in the cecal microbiota of chickens that *Alistipes* is a propionate producer expressing methylmalonyl-CoA epimerase, in which the gene for this enzyme is located on an operon with the acetyl-CoA carboxylase gene. It has also been shown that *Alistipes* is an acetate producer (59). Due to previous studies suggesting that short chain fatty acids (SCFAs) have anti-inflammatory mechanisms, it can be suggested that this decrease in *Alistipes* contributes to the decrease in SCFA and therefore contributes to the advanced fibrosis seen in these NAFLD patients.

Moreover, a study performed on mice with HCC showed the potential anti-inflammatory effects of a health-beneficial bacteria probiotic. It was believed that T-regs would be induced in patients with HCC thus suppressing the Th17 expression. A probiotic entitled Prohep was used, and after 38 days a reduction in tumor growth and Th17 cells was observed. Moreover, there was an increase in Treg/Tr1 cells, an anti-inflammatory cell subset. *Alistipes* was shown to increase in abundance in the mice cohort receiving the probiotic (45, 60). At the species level it is shown that *A. shahii* was one of the species that was significantly increased in the probiotic group. They hypothesize that *A. shahii* was playing a role in tumor suppression similar to what was is seen in cancer immunotherapy (61). Additionally, the investigators noted a significant increase in acetate and propionate metabolic potential in the probiotic group correlating with an increase in SCFAs-producing bacteria, such as *Alistipes* contributing to the suppression of Th17 cells in the gut, ultimately reducing the recruitment of Th17 cells to the liver (45). Also, in past studies it has been shown that SCFAs derived from fermented dietary fibers increase the levels of propionate found within the portal vein ultimately preventing cancer cell proliferation in liver tissue (62). Ultimately, *Alistipes* can be seen as a potential SCFA producer and their decrease contributes to these hepatic fibrotic conditions due to a decrease in anti-inflammatory cytokines and inability to suppress Th17 cells. Further studies will be important in the near future to better define the role of this genus in liver pathologies and health.

## *Alistipes* in Cardiovascular Disease, Hypertension, and the Epithelium

Cardiovascular disease (CVD) is the leading cause of mortality and morbidity in both developing and developed countries (5). With CVDs projected to rise in the future as the global population ages, the evaluation of the relationship with the gut microbiota has been investigated more extensively. *Alistipes* has been linked with CVD risk factors such as hypertension, as well as several CVDs such as atrial fibrillation (AF), congestive heart failure (CHF), and atherosclerosis cardiovascular disease (ACVD) (50).

In hypertension, it is believed that *Alistipes* contributes to inflammation and epithelium alterations. Kim et al. (48) demonstrated the relationship between the gut barrier dysfunction in patients with hypertension and the gut microbiota in humans. They used shotgun metagenomic analysis studying fecal samples from 22 patients with high blood pressure and 18 control patients. The data revealed an increase in *A. finegoldii* and *A. indistinctus* that was positively correlated with systolic blood pressure. Additionally, it was shown that *A. finegoldii*, known to trigger intestinal inflammation, had an increased number and functional genes in the high blood pressure cohort. Overall, the increase in *A. finegoldii* was observed to be positively correlated with systolic blood pressure (SBP), suggesting that this species is a potential driver for gut barrier dysfunction and inflammation in patients with high blood pressure. Also, it is believed that the hypotensive phenotype of inflammation is caused by the lipopolysaccharide (LPS) in *Alistipes*, which is known to be pro-inflammatory, leading to an increase in Th17 cells expressing CD161 and CCR6/integrin Beta7, as well as the decrease in butyrate-producing bacteria, which are known to be anti-inflammatory (47). Despite such argument, it is important to highlight that several other types of anaerobic bacteria, including species within the *Bacteroides* genus, may also contribute to the complexity of disease attributes conferred to date to the *Alistipes* genus. Further studies will be critical to elucidate more precise mechanisms.

Various studies have indicated that *Alistipes* plays a protective role in CVDs. Additionally, *Alistipes* has been associated directly with CVDs such as atrial fibrillation (AF). Atrial fibrillation is the most common arrhythmia and is prevalent in patients with hypertension, heart failure, and obesity (63). Atrial fibrillation is characterized on an EKG by the absence of P waves with irregular R-R intervals due to irregular beating of the atria leading to irregular conduction of impulses to the ventricles (64). In a study by Zuo et al. (16), conducted to quantify the relationship between the gut microbiome and AF, a whole-metagenomic shotgun sequence performed on 100 stool samples from Chinese participants, showed a drastic decrease in *Alistipes* spp. in the intestinal tract of patients with AF. However, bacteria that drastically increased during AF, such as *Streptococcus*, were proposed in the study to be the cause of the decline in *Alistipes*, suggesting a potential antagonistic effect between *Alistipes* and *Streptococcus*. This trend was commonly seen in other heart conditions, such as ACVD (50) and CHF (49). Evidence for the involvement of *Alistipes* in CVD is contradictory; that is, it is unclear if associations are protective or beneficial or pathogenic. Because most CVDs share common pathophysiological characteristics, such as endothelial dysfunction (16), it is possible that the role of *Alistipes* may depend on disease mechanisms shared across several CDVs. Therefore, more studies on the gut-heart axis could lead to the future understanding of microbiome-related diseases and potential therapies.

## *Alistipes* in Gut Inflammation and Other *BACTEROIDETES*

Due to the diverse microbial community in the gastrointestinal tract, there is a strong correlation between dysbiosis and inflammatory bowel disease (IBD). The most common IBDs within the human population are Crohn's disease (CD) and ulcerative colitis (UC). UC is a chronic inflammatory disease that primarily targets the colon. It has been suggested that *A. finegoldii* is a protective species against colitis since *A. finegoldii* is decreased in mice with colitis. Due to this fact, a study was performed in which microbiota-depleted mice were treated with oral DSS to induce colitis. When gavaged with *A. finegoldii*, the severity of colitis was similar to that of the WT mice (42). Mice developed colitis when *A. finegoldii* was added with *Bacteroides eggerthii*, a colitis- inducing bacteria, but the severity of the colitis was significantly decreased compared to that of mice with *B. eggerthii* alone or added with other bacteria such as *Parabacteroides distasonis*, or *Prevotella falsenii*. This further indicates that *A. finegoldii* is a colitis-attenuating bacteria (42). Of contrasting clinical interest, A. finegoldii has been isolated in association with other Bacteroides from gut-wall cavernous fistulous tract (CavFT) microlesions in severe Crohn's disease. The causal-effect and their prevalence in such lesions are currently under investigation in our laboratory (65–67).

A study by Butera et al. (68) showed 8 weeks old NOD2 knockout mice had an enrichment of *Alistipes*, anti-inflammatory cytokines (TGF-beta and IL-10), and CD4^+^LAP^+^FoxP3^−^ regulatory T cells. A possible connection for these observations comes from studies with curcumin, a spice that has been shown to modulate bowel inflammation by increasing CD4^+^LAP^+^FoxP3^−^ cells via IL-10 (69). To test to see the severity of the NOD2 knock-out in mice with colitis, they induced colitis via intra-rectal administration of 2,4,6-Trinitrobenzene sulfonic acid (TNBS) and used the expression of anti-inflammatory cytokine mRNA to determine the severity. It was found that the NOD2 knock-out mice had less severe colitis than the wild-type. Also, it was noted that the severity of the colitis was related to the different proportion of CD4^+^LAP^+^FoxP3^−^ cells observed prior to the TNBS treatment. Moreover, previous studies have shown a common trend of *Alistipes* abundance in NOD2 knock-out murine microbiota profiles (70). Of interest, *Alistipes* has been observed increasing among patients taking probiotics in an anti-inflammatory effect background (45). To date, it remains unclear what mechanisms of interaction exist between this genus and the other microorganisms in the gut, including food and probiotic strains, and the intramural fistulizing lessions we reported in surgical patients with CD. Metagenomic studies of fecal samples from the mouse model of spontaneous CD ileitis (SAMP1/YitFc, characterized by a fully penetrant 3D- stereomicroscopic pattern of segmental cobblestone ileitis resembling human CD) revealed an enrichment of Alistipes compared to the parental AKR/J mouse colony (cohabiting for >10 years in the same room), suggesting that Alistipes spp. could be associated with the promotion of segmental ileitis (65, 71, 72).

## *Alistipes* and Cancer via Beneficial Immunomodulation

Cancer, like cardiovascular disease, causes high rates of mortality and morbidity worldwide. Colorectal cancer (CRC) is one of the most common types of cancer typically targeting older individuals, as well as African Americans. CRC is also a form of cancer that has been linked to dysbiosis of the gut microbiota. *Alistipes* has been found to contribute to the pathogenesis, acting as a potential pathogen. Moschen et al. (15) showed that *A. finegoldii* promotes right sided colorectal cancer via the IL-6/STAT 3 pathway. Lipocalin 2 (LCN 2), an anti-microbial protein that binds to siderophores ultimately reduces iron availability (73). In patients with IBD LCN 2 is found in high concentrations in mucosal and fecal samples. Essentially, this can reduce the prevalence of *Alistipes*, as iron is a regulatory factor for the growth of *A. finegoldii*. However, Moschen et al. (15) went on to show that *A. finegoldii* caused intestinal inflammation after 1 week of being orally administered in WT, LCN 2 KO, and IL-10 KO C57BL/6J mice. Therefore, the paper concluded that *Alistipes* thrives in an inflamed environment that lacks LCN 2 and promotes inflammation and tumor formation. Moreover, they showed that *Alistipes finegoldii* was found in higher abundance in the ceca than other locations within the large intestine.

Despite the pathogenic effects observed for *Alistipes* in CRC, this genus has been shown to have a beneficial role in cancer immunotherapy by modulating the tumor microenvironment. One of the main hallmarks of cancers is to evade the immune system. Therefore, one form of anti-cancer treatment has been to manipulate the tumor microenvironment. An example of immunotherapy is to manipulate the microenvironment by inducing tumor necrosis factor (TNF) production by tumor-associated myeloid cells which ultimately leads to tumor eradication. One way to do this is to use a combination of intra-tumoral CpG-oligodeoxynucleotides (ODN) to activate TLR9, and inhibitory IL-10R antibodies. This immunotherapy typically halts tumor growth and induces TNF-dependent hemorrhagic necrosis by tumor-associated myeloid cells leading to tumor suppression.

In a study performed by Iida et al. (61) C57Bl/6 mice were injected subcutaneously with MC38 colon carcinoma cells and pre-treated with antibiotics (vancomycin, imipenem, and neomycin). The study initially determined whether antibiotics affected cancer immunotherapy. The authors found that antibiotics led to a decrease in efficiency of the tumor eradication due to a reduction in TNF production. The investigators then determined if those results depended on the bacterial load in the intestinal tract. Therefore, germ free (GF) mice with the MC38 tumors received anti-IL-10R/CpG-ODN treatment. Treated GF mice produced a significantly lower amount of TNF than specific pathogen free (SPF) mice. This suggests that the tumor-associated innate myeloid cells are primed by microbiota for inflammatory cytokine production in response to anti-IL-10R/CpG-ODN and that the reduced bacterial load from either the antibiotic treatment or germ-free status reduces this response and the TNF-dependent early-tumor necrosis. To better understand the role of antibiotics and the role of the gut microbiota, MC38 tumor bearing mice were gavaged with LPS and the TNF expression was reestablished. When they examined the microbiota involved via fecal microbiota composition, there was a positive correlation between *Alistipes* genus and a role for TLR4-priming/TNF production. Iida et al. (61) believed the TNF restoration was due to the role of pro-inflammatory gram-negative bacteria, *A. shahii*, binding to TLR4, priming the expression of TNF production. To further prove their hypothesis, authors then showed a delay in the recovery of *A. shahii* following antibiotic treatment, which also paralleled an ~4-week phase of TNF restoration after the antibiotic administration. Furthermore, they showed that when mice that were pre-treated with antibiotic and gavaged with *A. shahii*, the tumor-associated myeloid cells function to produce TNF was restored. Clinically relevant, the study indicated that when there is a reduction in *Alistipes*, there is a parallel reduction in optimal responses to cancer immunotherapy (61).

Others have also identified a role for *Alistipes* in cancer immunotherapy. For instance, non-small cell lung carcinoma (NSCLC) has a poor prognosis and no current therapy. Recently checkpoint inhibitors for PD-1 have been proposed as a potential immunotherapy for NSCLC. However, a major number of patients still persist with poor prognosis. Nivolumab is a fully human Immunoglobulin G4 monoclonal antibody against PD-1, therefore it blocks T cells from binding to the ligand PD-1L, typically expressed by the tumor, preventing T cell exhaustion. A study to find a potential correlation between gut microbiota and patients with NSCLC responding favorably to Nivolumab was conducted that presented evidence that *A. putredinis* was increased in patients who responded well to Nivolumab correlating with the study mentioned above (74).

## Mental Health

Although *Alistipes* can be found commonly in the intestinal tract, it has been shown to have a significant effect on diseases with localization outside of the gut such depression, anxiety, chronic fatigue syndrome, autism, cirrhosis, and aging. Dysbiosis within the intestine can affect the gut-brain axis and be used to explain the relationship between the gut microbiota, depression, and other mood disorders such as anxiety.

In a study conducted with BALB/c mice placed in a stressful environment, induced by grid-floor housing, there was a significant increase in *Alistipes* abundance (17). Furthermore, *Alistipes* concentration was also found to be increased, almost 4-fold, in Norwegian patients suffering from chronic fatigue syndrome (51). These findings correlate with the evidence of an increase in *Alistipes* for patients suffering from depression, since patients with depression typically struggle with fatigue and stress (75). It is believed that this increase in *Alistipes* disrupts the gut-brain axis because *Alistipes* is an indole-positive organism, and, thus decreases serotonin availability. Tryptophan is a precursor for serotonin, and a decrease in serotonin is associated with depression (52). Moreover, *Alistipes* has been shown to express glutamate decarboxylase, an enzyme that metabolizes glutamate into γ-aminobutyric acid (GABA) in chickens (58). This increase in *Alistipes* abundance could also possibly be related to the increase in GABA. However, studies should be done to show if the GABA is being secreted into the gut lumen (58).

Additionally, there are associations between the brain and gut amongst patients suffering from autism spectrum disorder. It is often found that individuals suffering from autism have frequent GI symptoms, speculating that it could be due to a dysbiosis within the microbiota. Strati et al. (53) found that there was a decrease in *Alistipes* in patients with autism spectrum disorder. However, another study done on a different form of autism, PDD-NOS, showed a significant abundance of *Alistipes* in children (54). It has been speculated that this could be from the production of propionic acid, which has shown to have neurobiological effects in rats (76). There is need for more studies on *Alistipes* and its effects on the gut-brain axis, since there is contradictory evidence regarding its protective/pathogenic role in both systems.

## Sulfonolipids and Biochemical Markers

*Alistipes* is a genus of bacteria with numerous immunological and biochemical pathways that are associated with the diseases mentioned above. One important implication is the promotion of CRC by *Alistipes* via IL-6/STAT 3 pathway. Thus, future studies could consider the use of *Alistipes* species as potential biomarkers for CRC, using our understanding based on microbiome DNA based data and the integration of biochemical concepts on disease pathogenesis. A potential method to accomplish this would be to look for sulfonolipids, a unique class of sphingolipids with a sulfonic acid group in the sphingoid base (77). Walker et al. (78) showed that when C57BL/6N mice are fed high fat diets with either safflower oil or lard fat for 3 weeks there is an increase in sulfonolipids, as well as, body weight when compared to mice fed the normal chow diet. A metagenome analysis was performed and screened for bacterial genes involved in sulfonolipid biosynthesis in the cecum of these mice. All species of *Alistipes* were found to produce sulfonolipids except *A. inops* (information on the most recent species A. *megaguti, A. provencensis*, and sulfonolipid production remain unknown). To further prove that sulfonolipids are a product of bacteria and a marker of *Alistipes*, scientists performed a mono-colonization study of germ-free (GF) mice with *Alistipes* spp. and detected a significant emergence of sulfonolipids in the cecum of the mono-colonized mice that was previously absent in the GF mice. Therefore, with common risk factors of CRC being high fat diets, obesity, and age (79), in addition to the increased abundance of *Alistipes* in these colonic conditions, there is the intriguing proposition for the potential of using sulfonolipids as markers for patients at risk for developing CRC, particularly within ethnicities, such as African-Americans with family histories of CRC. Additionally, studies should be conducted to verify if *Alistipes* abundance is increased in polyps, pre-cancerous vs. cancerous, being polyps another risk factor for CRC.

Moreover, *Alistipes* has the highest number of putrefaction pathways amongst commensal bacteria. Putrefaction is the fermentation of undigested proteins in the GI tract by the gut microbiota typically leading to bacterial production of harmful metabolites (80). These products have been reported as deleterious and associated with CRC (81). Such products include ammonia, H_2_S, cresol, indole, and phenol (82). In a study done to identify the main putrefaction pathways used by the gut bacteria, Kaur et al. (18), found that *Alistipes* contributed to histidine degradation/THF production, indole production, and phenol production. Histidine degradation/THF production have been found to release excess ammonia, which when absorbed, damages the colonic cells (83). Ammonia has also been found to increase intestinal cell proliferation and assist in the growth of cancer cells in CRC (84). Findings of excess ammonia and other *Alistipes* produced putrefaction products could be of use to clinicians when patients are at risk for developing CRC.

## Conclusion, Limitations, and Future Directions

**Figure 2:**
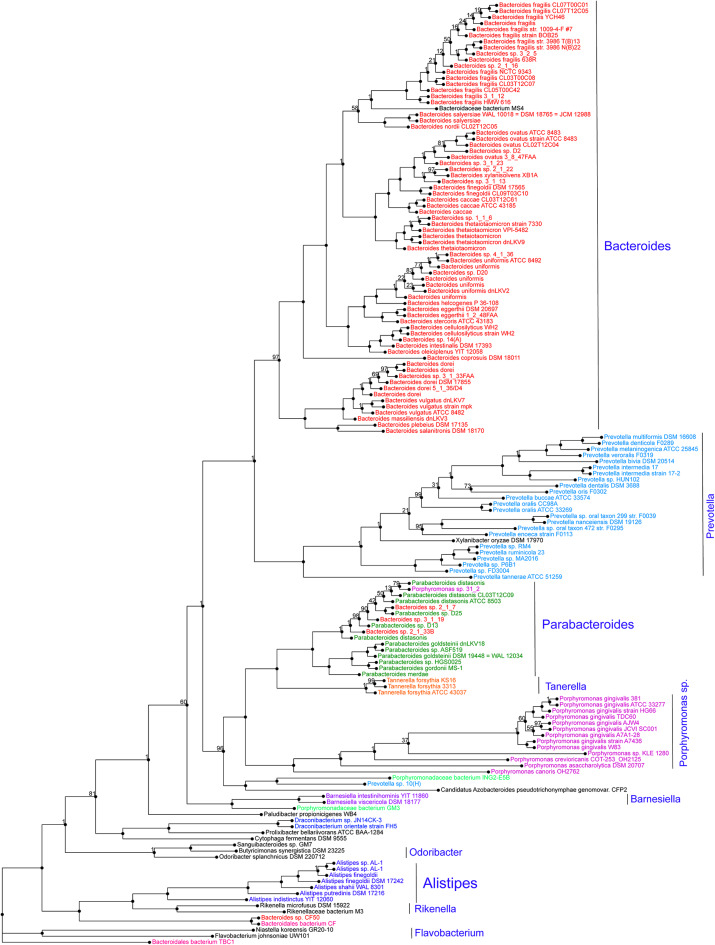
Protein phylogram of 155 complete genomes of the *Bacteroidetes* phylum to illustrate the potential functional distinction of the genus *Alistipes* within the group. The pipeline for genomic phylograms is described in detail as follow by PATRIC, the Pathosystems Resource Integration Center, https://docs.patricbrc.org. In short, the order-level pre-built trees in PATRIC are constructed by an automated pipeline that begins with amino acid sequence files for each genome. For each order-level tree the genomes from that order are used along with a small set of potential outgroup genomes. Branch values are not bootstrap values, which can be overly optimistic for long genomes. Instead, trees are built from random samples of 50% of the homology groups used for the main tree (gene-wise jackknifing). One hundred of these 50% gene-wise jackknife trees are made using FastTree, and the support values shown indicate the number of times a particular branch was observed in the support trees. As of May 19, 2020, there were 140 Alistipes genomes available (11287 unique contigs), of which 10 are complete.

*Alistipes* is a relatively new genus of bacteria isolated from clinical samples, although at a low rate compared to others within the *Bacteroidetes* phylum. At the protein level, a genome-wide protein phylogenetic analysis shown in Figure 2 illustrates that this genus may have unique functional properties that may enable them to have unique physiological roles, compared to other members within the *Bacteroidetes*, and/or also affect our ability to isolate these species *in vitro*. Therein, it is possible that the identification of this genus in clinical samples may be underrepresented as novel MS-TOF methods may not be fully capable to discriminate distinct species as separate. Immunologically, *Alistipes* has been seen to contribute to disease in both clinical and preclinical studies. Intriguingly, other studies have shown their presence is correlated with the promotion of healthy phenotypes such as *Alistipes* protective roles in diseases such as colitis, autism spectrum disorder, and various liver and cardiovascular fibrotic disorders. Despite *Alistipes* role in healthy phenotypes, *Alistipes* contrastingly has been shown to have a pathogenic role in diseases such as anxiety, myalgic encephalomyelitis/chronic fatigue syndrome, depression, PDD-NOS, and CRC.

Collectively, this review represents a summary of studies where *Alistipes* has been experimentally tested after inoculation into animal models, or where *Alistipes* has been found among other abnormally present species in human or animal microbiome studies. Thus, the perspective here presented, indicates that the genus, depending on the study discussed, could have a leading role in the modulation of diseases, or alternatively could just have either a bystander role, or a co-inducer role (with other gut microbes) of the observed clinical phenotypes. Animal studies will be further needed to decipher the mechanisms that may explain disease modulation alone and as symbiont by this genus across a multitude of complex multimodal diseases, which will benefit from the targeted study of subtype phenotypes, as we have analytically illustrated (9).

The use of germ-free animals and models will be beneficial to understand the role of this genus in disease and health and the interaction with the host immune defense tolerance, such as there should be studies investigating the roles on the SCFAs produced by *Alistipes* and their effect on the various liver diseases and *Alistipes* direct role on T-cell differentiation.

## Author Contributions

BP, PW, AV, and AR-P conceived the performed review and wrote the manuscript. All authors discussed and edited the manuscript and approved its final version.

## Conflict of Interest

The authors declare that the research was conducted in the absence of any commercial or financial relationships that could be construed as a potential conflict of interest.
